# Non-cognitive selected students do not outperform lottery-admitted students in the pre-clinical stage of medical school

**DOI:** 10.1007/s10459-015-9610-4

**Published:** 2015-05-03

**Authors:** Susanna M. Lucieer, Karen M. Stegers-Jager, Remy M. J. P. Rikers, Axel P. N. Themmen

**Affiliations:** Institute of Medical Education Research Rotterdam, Erasmus MC, Room AE-239, PO Box 2040, 3000 CA Rotterdam, The Netherlands; Department of Psychology, Erasmus University Rotterdam, Rotterdam, The Netherlands; Department of Social Sciences, University College Roosevelt, Utrecht University, Middelburg, The Netherlands; Department of Internal Medicine, Erasmus MC, Rotterdam, The Netherlands

**Keywords:** Academic performance, Cognitive selection, Control group, Medical school, Non-cognitive selection

## Abstract

Medical schools all over the world select applicants using non-cognitive and cognitive criteria. The predictive value of these different types of selection criteria has however never been investigated within the same curriculum while using a control group. We therefore set up a study that enabled us to compare the academic performance of three different admission groups, all composed of school-leaver entry students, and all enrolled in the same Bachelor curriculum: students selected on non-cognitive criteria, students selected on cognitive criteria and students admitted by lottery. First-year GPA and number of course credits (ECTS) at 52 weeks after enrollment of non-cognitive selected students (N = 102), cognitive selected students (N = 92) and lottery-admitted students (N = 356) were analyzed. In addition, chances of dropping out, probability of passing the third-year OSCE, and completing the Bachelor program in 3 years were compared. Although there were no significant differences between the admission groups in first-year GPA, cognitive selected students had obtained significantly more ECTS at 52 weeks and dropped out less often than lottery-admitted students. Probabilities of passing the OSCE and completing the bachelor program in 3 years did not significantly differ between the groups. These findings indicate that the use of only non-cognitive selection criteria is not sufficient to select the best academically performing students, most probably because a minimal cognitive basis is needed to succeed in medical school.

## Introduction

Medical schools all over the world select students as the number of applicants highly exceeds the number of available places. In Canada, the United States, the United Kingdom, and the Netherlands chances to be admitted to medical school range from 6 to 33 % (Razack et al. 2012) (https://www.duo.nl/particulieren/student-hbo-of-universiteit/loten/decentrale-selectie.asp). In addition, medical education is expensive for both student and society and medical schools have the responsibility of educating future medical doctors who are able to provide optimal healthcare (Hughes 2002). It is therefore important to select those applicants who will be able to successfully complete the program and will become well-performing medical doctors (Albanese et al. 2003; Basco et al. 2008; Burch 2009; Kulatunga-Moruzi and Norman 2002; Mitchell 1990).

A variety of selection instruments has been used to admit the most promising applicants to medical school. These instruments range from cognitive selection tools, like the pre-university grade point average (pu-GPA) (Ferguson et al. 2002; Siu and Reiter 2009) and aptitude tests such as the Medical School Admission Test (MCAT) (Albanese et al. 2003; Kelly et al. 2013) to selection instruments aiming to measure more non-cognitive capabilities of the applicants. Non-cognitive selection tools include regular interviews (Basco et al. 2008; Burch 2009), reference letters (Ferguson et al. 2003; Siu and Reiter 2009), motivation letters (Prideaux et al. 2011; Salvatori 2001), psychometric questionnaires (Arthur et al. 2001), Multiple Mini Interviews (Eva et al. 2004a, c), and Situational Judgment Tests (Lievens 2013; Patterson et al. 2012). The reliability and validity of these selection instruments differs widely, and while research has shown that mainly the pu-GPA and MCAT have, especially combined, good predictive value for performance during medical school (Albanese et al. 2003; Dunleavy et al. 2013; Ferguson et al. 2002), several non-cognitive selection tools appear not to be so reliable and valid (Basco et al. 2008; Burch 2009; Ferguson et al. 2003; Salvatori 2001). With respect to interviews, a large interviewer variability exists and personal factors such as gender and background of the applicant as well as expectations of the interviewer are sources of bias (Eva et al. 2004b; Salvatori 2001). Problems with reference and motivation letters are the absence of a scoring system to compare the letters (Ferguson et al. 2002) and that they rarely distinguish one applicant from another (Spina et al. 2000). The use of psychometric questionnaires for selection procedures is also criticized, because of the possible lack of reliability and the risk of obtaining desirable answers (Arthur et al. 2001). Although the first results on the MMI and SJT are promising (Dore et al. 2010; Eva et al 2004c; Koczwara et al. 2012), further research is needed to establish the long-term predictive value of these non-cognitive selection tools.

Whereas much research has been conducted on the predictive validity of selection tools, only a few studies included a control group to compare the performance of the selected students to (Urlings-Strop et al. 2013; Urlings-Strop et al. 2009; 2011). In addition, the predictive value of the selection tools might also depend on the curriculum employed by the medical school (Edwards et al. 2013), which limits the generalizability of the findings and the possibility to compare the selection methods. It would thus be valuable to determine the independent contribution of non-cognitive and cognitive selection methods to academic performance in the presence of a control group, allowing a comparison of all groups under the similar circumstances.

While many medical schools in the world select 100 % of their applicants, the situation in the Netherlands is different, as at the time of the current study maximally 50 % of the students were selected by the medical schools (Urlings-Strop et al. 2011), although the percentage of selected students is expected to rise also in the Netherlands in view of recent policy changes phasing out the lottery practice (http://www.rijksoverheid.nl/nieuws/2014/08/30/centrale-loting-afgeschaft.html; in Dutch).

In the Netherlands, applicants currently have three possibilities to be admitted to medical school: direct access, selection, and lottery. Direct access is provided to those applicants who obtained a pu-GPA of ≥8.0, on a scale from 1 to 10, where students need to score at least a 5.5 to pass. As regards to the students admitted by selection, each medical school employs its own particular, local selection procedure. Finally, non-selected students are admitted via a national lottery system that is weighted for secondary school performance, i.e. applicants are assigned to a lottery batch depending on their pre-university grade point average (pu-GPA), with increasing odds to be admitted with increasing pu-GPA. Applicants who failed to be admitted by the local selection procedure are allowed to participate in the national lottery, which means they still have a chance to enter medical school. This provides a unique opportunity to create a control group of non-selected, but lottery-admitted medical school students.

A few studies have already taken advantage of this situation and studied the effect of selection on academic performance (Hulsman et al. 2007; ten Cate et al. 2002; Urlings-Strop et al. 2009, 2011, 2013). However, some of these studies were conducted during the development of procedures when selection for medical school was first allowed in the Netherlands, resulting in a relatively small number of selected students (1.5–6.2 %), which might have influenced their findings (Hulsman et al. 2007; ten Cate et al. 2002). These studies showed that selected students were more committed to health care during their first 2 years of medical school but did not perform better (Hulsman et al. 2007) and showed that cognitive requirements do have some predictive value for academic performance (ten Cate et al. 2002). Research conducted at our own medical school showed that a selection procedure existing of a non-cognitive step followed by a cognitive one led to the inclusion of students who received higher grades during clerkships and dropped out less often than students who were admitted by lottery (Urlings-Strop et al. 2009, 2011). In addition, one of the studies by Urlings-Strop et al. (2013) showed that success in a cognitive selection step related to a lower dropout rate, while higher grades during clerkships related to success on non-cognitive selection criteria. However, here, only those applicants who met the non-cognitive requirements were allowed to participate in the following cognitive step of the selection procedure, which made it impossible to measure the effect of cognitive or non-cognitive selection in isolation. A final study is from researchers in Denmark. They investigated the difference in dropout between students who were admitted on either their pu-GPA or on their results in a selection procedure. In this selection procedure, applicants were judged upon motivation, qualifications, general knowledge, and performance in an admission interview. This study showed that students who were selected dropped out less often compared to the students who were admitted on the basis of their pu-GPA (O’Neill et al. 2011). However, this study only focused on dropout and not on other measures of academic performance.

In the current study, we aimed to examine the effect of either non-cognitive or cognitive selection in isolation on academic performance using the lottery-admitted group as a control. In this manner the contribution of non-cognitive selection criteria and cognitive selection criteria could be measured independently among students who followed the same curriculum and could be compared to the lottery-admitted students.

We expect all selected students to drop out, voluntary or academically dismissed, less often than the students who were admitted by lottery, as previous studies showed that students who had to put effort in their admission, dropped out less often, irrespective of their success in the selection procedure (O’Neill et al. 2011; Urlings-Strop et al. 2013). In addition, we expect the non-cognitive selected students to pass the third-year objective structured clinical examination (OSCE) more often than the other admission groups since previous research showed that success on non-cognitive criteria was related to higher clerkship grades (Urlings-Strop et al. 2011). Next to this, we were interested to investigate whether there were other differences in academic performance, e.g., grade point average and number of course credits, between the non-cognitive selected students, the cognitive selected students and the lottery-admitted students.

## Methods

### Context

This study was performed at the Erasmus MC Medical School in Rotterdam, the Netherlands. Everyone who has finished a pre-university secondary school level, with a combination of subjects obligatory for medicine, or who has similar qualifications, is allowed to apply to medical school in the Netherlands (Ten Cate 2007).

Normally, in the local selection procedure of the Erasmus MC Medical School, applicants are selected if they succeed in two, consecutive, selection rounds: a non-cognitive (i.e., quality and quantity of their extracurricular activities before application) and a cognitive round (i.e., their scores on a set of five cognitive tests covering a medical subject).The cognitive round was limited to those who met a cutoff value in the first, non-cognitive, round. As regards to the extracurricular activities, applicants must have spent at least 4 h per week for a minimum of 1 year on (a) voluntary work-related activities in healthcare, (b) a managerial position in for example a school board or (c) have achieved an outstanding performance in sports, science, literature or art. The rationale behind selecting applicants upon their extracurricular activities is that these applicants are able to distinguish themselves from others, by showing through their behavior the motivation and ambition to carry out other activities as well as the ability to combine these activities with their secondary education (Urlings-Strop et al. 2009). In the cognitive step, the applicants’ level of cognitive ability and academic study skills was measured by their performance on five different cognitive tests. Test subjects include arithmetic’s, anatomy, scientific reading, logical thinking, and one test based on a medical subject referring to two lectures applicants have attended.

Once students are admitted to medical school, they follow an integrated and theme-oriented curriculum that comprises a 3-year Bachelor degree course followed by a 3-year Master degree course. The Bachelor of Medicine is divided into thematic blocks of 4–16 weeks, which are organized around pathophysiological systems and cover subjects starting from the basic sciences up to and including clinical practice. Per academic year, students can achieve a maximum of 60 European Credits (ECTS). ECTS are a standard for comparing student workload in the European Union and reflects successfully completed exams and assignments (Kuncel et al. 2014). Students who quit voluntary within the first 2 years of enrolment, or who do not meet the requirements set by medical school, i.e. having earned all first year ECTS by the end of the 2nd year of enrollment, are considered as dropouts.

### Participants

In the present study, we included students who started in 2008 and in 2009 at Erasmus MC medical school, and were admitted by selection or lottery. Those who were directly admitted based upon their pu-GPA (≥8.0) were excluded from this analysis since these students have not participated in either selection or lottery and their numbers are small (<10 %).

In total, 550 students (*M*_*age*_ = 19.4, *SD* = 1.5) were included; 102 non-cognitive selected students, 92 cognitive-selected students and 356 lottery-admitted students. No significant differences in age, gender and pu-GPA were found between the admission groups and cohorts (see Table 1).

**Table 1:** Descriptive statistics per admission group and per cohort

	N	Mean age (*SD*)	% Female	Mean pu-GPA (*SD*)
2008				
Non-cognitive selected students	102	19.3 (1.5)	64.7	6.9 (.50)
Lottery-admitted students	190	19.4 (1.2)	63.2	7.0 (.51)
Total 2008	292	19.3 (1.3)	64.6	7.0 (.51)
2009				
Cognitive selected students	92	19.6 (2.0)	51.5	7.1 (.53)
Lottery-admitted students	166	19.5 (1.4)	61.4	7.0 (.53)
Total 2009	258	19.5 (1.6)	57.8	7.0 (.53)
Total lottery-admitted students	356	19.4 (1.3)	62.4	7.0 (.52)
Total	550	19.4 (1.5)	60.9	7.0 (.52)

*SD* standard deviation

### Procedure

For the experiment in this study, the regular local selection procedure of the Erasmus MC Medical Center was adapted. While normally applicants are admitted when they succeed in both consecutive rounds, for this experiment, only the score in one round was taken into account. During 1 year, students were admitted only on the basis of their extracurricular activities (i.e., the non-cognitive selected students). They did participate in the cognitive step, but these results did not influence the decision to admit them to medical school. In the following years’ selection, all students were admitted solely on the basis of their test scores (i.e., the cognitive selected students). They still had to hand in information about their extracurricular activities but were all allowed to take the cognitive tests, independently of the quality or quantity of their activities. In addition, in both years, at least 50 % of the places were available for students who participated in the national lottery system. From these three different admission groups, dropout and various measures of academic performance were determined.

### Dropout and measures of academic performance

Dropout was defined as quitting voluntary within the first 2 years, or failing to acquire all 60 ECTS of the first-year subjects within the first 2 years of enrolment, as the latter one is a requirement to continue the program. Academic performance was measured by (1) the mean grade of the exams in first-year at first attempt (first-year GPA), (2) the number of ECTS at 52 weeks of enrollment, (3) passing the third-year OSCE and (4) completing the Bachelor course in 3 years (i.e., having obtained 180 ECTS in 3 years).

### Statistics

Data were analyzed with IBM SPSS AMOS 18.0 (SPSS, Inc., Chicago, IL, USA). Since different cohorts were included in this analysis, first-year GPAs were converted into z-scores. ANOVA with post hoc Bonferroni tests were used to compare the first-year GPA and ECTS at 52 weeks of the non-cognitive selected students, the cognitive selected students, and the lottery-admitted students. Since first-year GPA and the number of ECTS at 52 weeks were not normally distributed, *Welch F* was calculated. Effect sizes were determined using eta square with values of .01, .06, and .14 indicating small, medium and large effects (Cohen 1988; Lakens 2013). Odds ratios (OR) with Wald statistics were obtained from logistic regression analyses to determine the chance of passing the OSCE, completing the Bachelor program in 3 years and the chance of dropping out, for all three admission groups.

### Ethical considerations

In the present study, all data were processed anonymously to make sure that no possible harm to participants could arise from this study. Since medical school are allowed to set their own selection policies, the methods employed in the present paper did not constitute an experiment with respect to the applicants. The data on academic performance used in this study were collected as part of regular academic activities and obtained from the university administrative system, no individual informed consent was required.

## Results

No statistically significant differences were found between the non-cognitive selected, the cognitive selected, and the lottery-admitted students in first-year GPA, but cognitive-selected students had earned significantly more ECTS at 52 weeks than the lottery-admitted students (See Table 2). In addition, cognitive-selected students had a significantly lower probability to drop out than lottery-admitted students (11 % vs. 21 %). The probabilities to pass the third-year OSCE and to obtain 180 ECTS in 3 years did not significantly differ among the three admission groups (Table 2).

**Table 2:** Comparison of academic performance of non-cognitive selected, cognitive selected and lottery-admitted students by ANOVA and odds ratios

	GPA, mean z score ± SD	ECTS at 52 weeks ± SD	OSCE OR (95 % CI)	Drop-out OR (95 % CI)	Bachelor degree in 3 years OR (95 % CI)
Non-cognitive selected students	.078 ± .87	48.1 ± 18.4	1.22 (.73–2.04)	.97 (.42–2.22)	1.51 (.88–2.57)
Cognitive selected students	.185 ± .86	51.8 ± 14.3	.886 (.50–1.57)	.32 (.14–.77)	1.11 (.66–1.86)
Lottery-admitted students	−.071 ± 1.06	44.8 ± 20.4	1.00 (–)	1.00 (–)	1.00 (–)
Test value	*Welch F* = 1.765	*Welch F* = 7.418**		*Wald* = 6.557*	
Effect size		*η* ^2^ = .019			

*SD* standard deviation, *OR* Odds ratio, *OSCE* objective structured clinical examination, 95 % CI *=* 95 % confidence interval

* Significant at *p* = .01, ** significant at *p* < .01, η^2^ = effect size for ANOVA, with .01, .06, and .14 indicating small, medium and large effects

## Discussion

The aim of this study was to gain insight in the effect of different selection criteria on academic performance. We therefore set up a study that enabled us to compare the academic performance of non-cognitive selected students to cognitive selected students, and to lottery-admitted students. This study is, to our best knowledge, the first to compare two groups of students selected using different types of criteria, who all take part in the same curriculum, and which also involves a control group (i.e., the lottery admitted students).

Our hypothesis, that both groups of selected students would drop out less often than the lottery-admitted students, was partly confirmed. Although non-cognitive selected students did not drop out less often than lottery-admitted students, cognitive selected did. Contrary to our expectations, non-cognitive selected students did not pass the OSCE more often than cognitive-selected and lottery-admitted students. We did however find a difference in first-year performance between the admission groups: cognitive selected students earned more ECTS in 52 weeks than lottery-admitted students.

It was somewhat surprising that non-cognitive selected students did not drop out less often than lottery-admitted students. Previous research showed that participating in a selection procedure, irrespective of the outcome, prevented students from dropping out. Put differently, students who were willing to put effort in their admission were shown to drop out less often (O’Neill et al. 2011; Urlings-Strop et al. 2013), which is contrary to our findings. A possible explanation for this outcome is that motivation and ambition, the characteristics that were aimed to be determined in the non-cognitive round, are by themselves not enough to succeed in medical school. Indeed, research has shown that highly motivated students with inadequate and ineffective study methods have a higher chance of dropping out (Bennett 2003), and that students also need to be able to adapt to the academic environment in order to perform well (Stage 1989).

Another explanation for our contrary finding that might be interesting to investigate in future research, is whether the rationale behind the applicants’ behavior has changed over the years. That is, the requirements for admission have been made more transparent since the start of selection for medical school in the Netherlands, and applicants are much more aware of what is expected of them. The original reasoning behind the use of extra-curricular activities for selection was that applicants with many extra-curricular activities showed this behavior consistent with their motivation or ambition to employ other activities (Urlings-Strop et al. 2009). It may be the case that applicants nowadays invest time in extra-curricular activities just because they want to enter medical school and are less driven internally. Thus, the quality of the motivation and ambition behind their behavior may have changed resulting in a diminished positive effect on drop out.

The finding that the non-cognitive selected students do not pass the OSCE more often than the cognitive selected and lottery-admitted students was also unexpected, since previous research showed that the non-cognitive selection step was related to higher clerkship grades (Urlings-Strop et al. 2011). It has, however, to be mentioned that only one study examined this relationship so far. Thereby, the results on the OSCE are not very discriminative since they are binary, you either pass or fail the test. Other criteria, or even a more discriminating OSCE, may be more relevant in judging performance in different stages of medical education. Future research is needed to investigate whether the non-cognitive selected students in this study do indeed earn higher clerkship grades. Apart from this, one could argue that the non-cognitive selected students did not pass the OSCE more often since a certain level of cognitive ability is required for optimal clinical performance. Several researchers acknowledged that just being able to communicate to a patient is not sufficient, since the conversation is less effective when the necessary knowledge is absent (Eva et al. 2009; Miller 1990). Multiple studies therefore encourage the use of a combination of pre-university performance or cognitive measures as well as non-cognitive tests when selecting future medical doctors (Ferguson et al. 2003; Ranasinghe et al. 2012), and also the findings in this current study indicate that selection on only non-cognitive requirements is not sufficient. Non-cognitive criteria thus should be accompanied by cognitive selection criteria, since someone needs to have a sufficient level of cognitive ability as well to succeed in medical school.

The final outcome of this study, that cognitive selected students do drop out less often and earn higher grades or more ECTS in 52 weeks than the lottery-admitted students, is less surprising, since our Bachelor program is mainly cognitive based. These findings indicate that the cognitive selection criteria set a cognitive standard that students need to have to perform well in medical school. One could argue that, in the Netherlands, a cognitive standard is set by allowing only those applicants in medical school who have completed the highest level of secondary education. Nevertheless, there are differences between secondary school and university that might result in the fact that not everyone who is able to pass secondary education, performs well in medical school. For example, while secondary schools exams focus on one course, medical school examinations cover multiple domains, ranging from basic sciences to clinical knowledge. Therefore, different knowledge and study skills are required to pass the exams. Since the cognitive tests used in the selection procedure resemble medical school examinations and aim to access the applicants’ academic study skills, their results provide incremental validity on top of the secondary school examinations.

This study is not without limitations. In the Netherlands, a very homogeneous group of students applies for medical school, since different tracks (vocational, pre-higher education) of secondary education exist and students are at the start of secondary education (age approximately 12 years) matched to a level that suits their competences. Only those students who have finished the highest level of secondary school, a pre-university secondary school level with a combination of subjects adequate for medicine, are allowed to apply to medical school (Ten Cate 2007). Thus, almost all applicants for medical school share many characteristics; they all graduated at the highest level of secondary education, all followed the same subjects and they all have the ambition to study medicine. Selection in homogeneous groups is difficult to achieve since clear cutoffs between who is selected and who is not are hard to define, resulting in little variation in outcome scores. The differences in academic performance between those who are and those who are not selected, may be more apparent in a more heterogeneous group where more variation in selection scores is visible and better defined cutoffs can be applied. Another limitation concerns the use of the OSCE in year 3, the strength of this outcome measure is diminished by its dichotomous nature and by student dropout, limiting student completion. A final limitation is that the Dutch lottery system uses pu-GPA weighted odds and therefore the lottery-admitted students do not constitute an ideal, random control group. Nevertheless, their age, gender and pu-GPA did not differ from the admission groups and between the cohorts.

This study provides an important practical implication for medical schools. Most medical schools have to select, since the number of applicants is exceeding the number of places available (Razack et al. 2012), and even though the importance of non-cognitive selection is widely acknowledged (Hughes 2002; Hurwitz et al. 2013) the outcomes of this study indicate that medical schools should include at least some cognitive requirements, besides non-cognitive measures, in their selection procedure in order to admit the most promising students in medical school. Such cognitive tests should ideally measure knowledge and academic study skills required for medical school, as for example the ability to obtain deep understanding of complex subjects by integrating information from various sources such as lectures, scientific papers and self-study. This way, the cognitive tests provide incremental validity over high school.

In addition, our findings show that selection of non-cognitive characteristics alone has no predictive value for success in medical school, suggesting that non-cognitive selection should be accompanied by some type of cognitive measure, such as pu-GPA, aptitude tests or study skill determination.

## Conclusion

This study showed that students who were selected on non-cognitive criteria do not outperform lottery-admitted students during the Bachelor years of medical education, but students who were selected on cognitive criteria did earn more ECTS in the first 52 weeks and dropped out less often than lottery-admitted students. Our findings suggest that non-cognitive selection is not sufficient by itself but should be accompanied by cognitive selection, since a certain cognitive basis is required to succeed in medical school.
